# Effects of medium-term green tea extract supplementation combined with CrossFit workout on blood antioxidant status and serum brain-derived neurotrophic factor in young men: a pilot study

**DOI:** 10.1186/s12970-019-0280-0

**Published:** 2019-03-21

**Authors:** Ewa Sadowska-Krępa, Przemysław Domaszewski, Ilona Pokora, Aleksandra Żebrowska, Agnieszka Gdańska, Tomasz Podgórski

**Affiliations:** 1 Department of Physiological and Medical Sciences, the Jerzy Kukuczka Academy of Physical Education, Katowice, Poland; 2 Department of Tourism and Health Promotion, University of Technology, Faculty of Physical Education and Physiotherapy, Opole, Poland; 3 Department of Biochemistry, Poznan University of Physical Education, Poznań, Poland

**Keywords:** Green tea extract, Prooxidant-antioxidant balance, BDNF, CrossFit

## Abstract

**Background:**

Potential health benefits are attributed to the antioxidant properties of green tea polyphenolic compounds. The main aim of the study was to evaluate the effects of a six-week green tea extract (GTE) supplementation combined with CrossFit workout on blood antioxidant status and serum brain-derived neurotrophic factor (BDNF) in men.

**Methods:**

Sixteen young males involved in CrossFit training were randomized into two groups supplemented with GTE or placebo for six weeks. Each participant performed an exercise test for the evaluation of maximum oxygen uptake (VO_2_max) twice, i.e., before starting (1st trial) and after completing the supplementation combined with CrosFit workout (2nd trial). Venous blood samples were drawn at rest, immediately post-test and after one hour of recovery in order to estimate activities of antioxidant enzymes (superoxide dismutase [SOD], catalase [CAT], glutathione peroxidase [GPx], reduced glutathione [GR]), non-enzymatic antioxidants (reduced glutathione [GSH], uric acid [UA], total phenolics), total antioxidant capacity (FRAP), lipid peroxidation products (TBARS), and BDNF.

**Results:**

Except for a significantly higher SOD activity and FRAP level recorded at rest and post-exercise in the 2nd trial compared to the corresponding values in the 1st trial, no significant differences were recorded among other assayed measures such as CAT, GPx, GR, GSH and BDNF. Moreover, a percentage increase in FRAP level was twice as high after six weeks’ GTE consumption than after placebo. Regardless of the trial, an increase in plasma UA concentration and a decrease in plasma total phenolics level were observed after exercise test. Plasma TBARS concentrations were significantly higher in PLA group after six weeks’ CrossFit training, while in GTE group they were slightly lower compared to the corresponding values in the 1st trial. Moreover, there was a significant inverse correlation between FRAP and TBARS in the GTE-supplemented group (*r* = − 0.40, *p* < 0.05).

**Conclusions:**

A six weeks’ consumption of GTE had marginal effect on aerobic capacity and serum BDNF level in CrossFit-trained men, but it caused a marked increase in the blood antioxidant capacity and a moderate attenuation of the training-induced lipid peroxidation.

## Background

Numerous studies have revealed that regular consumption of green tea provides protection against diabetes, cardiovascular disease, cancer, infection, and brain-specific disorders [1–3]. Its potential health benefits are mainly attributed to the antioxidant properties of polyphenolic compounds containing the phenolic hydroxyl groups attached to ring structures through acting as hydrogen- or electron-donating free radical and transition metal chelators. Moreover, they can inhibit the activity of enzymes responsible for reactive oxygen species (ROS) production, such as xanthine oxidase, cyclooxygenase and lipoxygenase [4].

The health effect of polyphenols is associated with their bioavailability. Before intestinal absorption, these compounds are hydrolyzed by enzymes or by colonic microflora and then in the liver where they are conjugated via methylation, sulfation or glucuronidation [5]. Polyphenol metabolites reach the blood and then transferred to various body tissues and organs such as the brain [2, 3]. Paradoxically, polyphenols are also known to have prooxidant properties both in vitro and in vivo; it can therefore be presumed that their biological effects are not limited to modulation of oxidative stress [6].

Green tea extract is known for its high flavonoid content, including catechins and epicatechins, with epicatechin (EC), epicatechin-gallate (ECG), epigallocatechin (EGC) and epigallocatechin 3-gallate (EGCG) being the most abundant (about 50–75%) by weight [6]. Green tea polyphenols can act as direct antioxidants by scavenging ROS in vitro, as well as acting indirectly by potentially up-regulating phase II antioxidant enzymes [7]. The rate of their ability to quench free radical species was found to increase in the following order: EC ≤ ECG < EGC ≤ EGCG [8]. Of catechins, EGCG is the most bioactive and its role as an antioxidant relies on modulating antioxidant defense and preventing oxidative damage in healthy cells [9, 10]. Of note, EGCG could have been responsible for increase in maximal oxygen uptake in adult humans [11]. On the other hand, EGCG may have prooxidant effects in vivo and lead to hepatotoxicity at high oral doses [12]. Chronic treatment with EGCG in doses from 700 to 2100 mg/d has been found to lead to elevations in serum transaminase and bilirubin levels, and abdominal pain [13].

It has been shown that neurodegenerative diseases are closely associated with a neuronal dysfunction and a low level of brain-derived neurotrophic factor (BDNF) in brain regions including the hippocampus [14]. BDNF plays an important role in long-term potentiation of learning and memory [15]. According to Gómez-Pinilla and Nguyen [2] antioxidant properties of EGCG, as a natural catechin polyphenol, can be associated with an increased expression of BDNF and higher cognitive function, better mood, and protective effects against various brain diseases. Therefore, using green tea polyphenols would be a highly useful complementary approach for an inexpensive long-term treatment of neurodegenerative diseases, particularly due to a synergic interaction observed between green tea polyphenols, as a result of EGC- and EC-promoted action of EGCG [16]. Moreover, physical exercise promotes an increase of BDNF in the brain [17].

CrossFit is a popular multimodal form of high intensity training including a wide range of physical activities such as running, swimming, paddling, weightlifting, and gymnastics. The most physical exercises are performed in a circuit format with a short rest or without rest in order to improve aerobic and anaerobic endurance, speed, flexibility, agility, motor coordination and body shaping [18, 19]. It is well known that the CrossFit training program beneficially affects processes leading to improved well-being [20]. In turn, other studies have shown that CrossFit can cause training-induced oxidative stress and lead to disturbance of the prooxidant-antioxidant balance [21].

To our knowledge there is no information as to how a medium-term consumption of green tea extract combined with CrossFit workouts affects blood antioxidant status and neuroprotective effects. Therefore, the aim of this study was to examine whether six weeks’ daily consumption of green tea extract combined with CrossFit workouts would affect aerobic capacity, blood antioxidant status and serum BDNF level in trained men.

## Methods

### Participants

Sixteen male students of the Physical Education Faculty volunteered to enter the study. Our research included 20 subjects, however, only sixteen of them were able to complete the study.

All individuals received information on the aim and nature of the study and gave their written consent to participate. The study conform to the ethical guidelines of the Declaration of Helsinki and received approval of the Local Bioethics Committee (certificate of approval No. 4/2013). The exclusion criteria included tobacco use, alcohol consumption and medication/dietary supplements during the four weeks preceding the study.

All students involved in a six-week CrossFit workout were randomized into two groups: 1) a control placebo group (PLA, *n* = 8) and 2) a group supplemented with green tea extract (GTE, n = 8). The intake of GTE or PLA supplements was controlled. The basic physical characteristics of subjects are presented in Table 1.

**Table 1:** Basic characteristics of the participants in response to GTE supplementation and CrossFit workout

Group	Age (years)	Height (cm)	Body weight (kg)	VO_2_max (ml/min/kg)	
				1st trial	2nd trial
PLA	23.1 ± 1.7	180.0 ± 8.3	80.1 ± 11.5	45.5 ± 5.4	47.3 ± 5.4
GTE	22.0 ± 1.1	181.6 ± 6.2	77.6 ± 6.4	45.6 ± 5.6	48.8 ± 4.6

Data are provided as mean ± standard deviation (SD); *Abbreviations*: *PLA* the control placebo group, *GTE* the supplemented with green tea extract group, *VO*_*2*_*max* maximal oxygen uptake.

### Supplements

Supplements in the form of soft gelatinous capsules (Olimp Labs, Dębica, Poland) were administered at a dose of two capsules once daily for six weeks. Placebo capsules contained microcrystalline cellulose, magnesium stearate and maltodextrin instead of plant extract. One GTE capsule contained 250 mg of standardized green tea extract (245 mg polyphenols, including 200 mg catechins, among them 137 mg epigallocatechin-3-galate and additional substances such as caffeine < 4 mg, microcrystalline cellulose, and magnesium stearate).

### CrossFit workout

The six-week CrossFit program was based on the CrossFit training guide [22] and consisted of workouts of the day (WOD). It was scheduled by a professional certified coach (CrossFit, Inc., Berlin, Germany) according to a five-day-on and two-day-off pattern. Each training unit started with a warm-up, took about 50 min and finished with an intense stretching of the whole body. The workouts are composed of three distinct modalities: monostructural metabolic conditioning (M), gymnastics (G), and weightlifting (W) [23]. During each training unit the workouts were represented by involving one, two or three modalities.

The main purpose of metabolic conditioning was to improve cardiorespiratory capacity and stamina by performing exercises such as running, biking or rowing. The gymnastics modality included air squats, pull-ups, push-ups, dips, handstand push-ups, rope climbs, muscle-ups, presses to handstand, back/hip extensions, sit-ups, and jumps leading to improved coordination, balance, agility, and accuracy. The weightlifting modality, which included deadlifts, cleans or presses, led to an increase in strength and power (see Table 2).

**Table 2:** A five-day-on and two-day-off workout structure of CrossFit

Week/day	CrossFit workout
1st week	
1st day (M)	for time: 5 x 400 m run/ rest 1 min/ 5000 m run
2nd day (G,W)	for time 21-15-9 reps: thrusters 42,5 kg/ pull-ups
3rd day (M,G,W)	for time: 50 push-ups/ 50 sit-ups/ 50 deadlifts (60 kg)/ 50 wall ball shots (10 kg)/ 50 box
4th day (M,G)	20 minute AMRAP: 400 m run/ max reps pull-ups
5th day (W)	muscle clean (5 x 1 rep)/ power clean (5 x 3 reps)/ clean (5 x 5 reps)
6th & 7th day	Off
2nd week	
1st day (G)	skill and progression: muscle-up
2nd day (M,W)	4 rounds for time: 50 kcal row/ 30 front squat (40 kg)
3rd day (M,G,W)	13 min AMRAP: 40 double unders/ 20 toes to bar/ 10 over head squat (40 kg)
4th day (G, W)	8 min AMRAP/ 1 min. rest/ 8 min AMRAP: 5m rope climb/ 6 snatch (40 kg)
5th day (M)	3 rounds for time: 50 steps over head lunges (15 kg)/ 200 run
6th & 7th day	Off
3rd week	
1st day (W)	muscle snatch (5 x 1 rep)/ power snatch (5 x 3 reps)/ snatch (5 x 5 reps)
2nd day (M,G)	5 rounds for time: 30 double unders/ 6 hand stand push-ups/ 20 sit-ups/ 10 burpee box jumps
3rd day (M,G,W)	3 rounds for time: row 100 kcal/ 50 pull-ups/ 15 sumo deadlift high pull (35 kg)
4th day (M,W)	1 km run/ front squat 6-6-4-4-2-2-1-1 reps
5th day (G)	10 rounds for time: 3 weighted pull-ups (15 kg)/ 5 strict pull-ups/ 7 kipping pull-ups
6th & 7th day	Off
4th week	
1st day (M)	5 rounds for time: 1 km run/ rest 2 min
2nd day (G,W)	20 min muscle-ups: 3-6-9-12-9-6-3 reps: 10 min max reps over head squat (40 kg)
3rd day (M,G,W)	for time: 1 km row/ 50 thrusters (30 kg)/ 30 pull-ups
4th day (M,G)	for time: 100 pull-ups/ 100 push-ups/ 100 sit-ups/ 100 squats
5th day (W)	medicine ball clean; snatch balance 5-5-5-3-3-1-1 reps/ snatch 3-3-2-1-1 reps
6th & 7th day	Off
5th week	
1st day (G)	skill and progression: hand stand
2nd day (M,W)	5 rounds for time: 400 m run/ 15 over head squats (40 kg)
3rd day (M,G,W)	for time: 50 box jumps/ 50 push-ups/50 kettlebell swings/ 50 walking lunges/ 50 burpees
4th day (G, W)	skill and progression: turkish get-up; max load back squat 1-1-1-1-1 reps
5th day (M)	1000 burpees
6th & 7th day	Off
6th week	
1st day (W)	for time: snatch (60 kg) 30 reps
2nd day (M,G)	5 min AMRAP: 15 double unders/ 5 strict pull-ups; 5 min AMRAP: 100 m run/ 5 push-ups
3rd day (M,G,W)	20 min AMRAP: 5 hand stand push-ups/ 10 pistol squats/ 15 pull-ups
4th day (M,W)	5 rounds for time: 5 deadlifts (120 kg)/ 10 burpees/ 1 min rest
5th day (G)	skill and progression: bar muscle-ups
6th & 7th day	Off

*Abbreviations: M* metabolic conditioning, *G* gymnastics, *W* weightlifting, *AMRAP* as many rounds as possible

The influence of CrossFit training on aerobic capacity was assessed based on incremental cycle ergometer testing (Sport Excalibur, Groningen, The Netherlands) during which the participants were connected to a breath-by-breath gas analyzer (MetaLyzer 3B-R2, Leipzig, Germany). Maximal oxygen uptake (VO_2_ max) was determined prior to (first trial) and after 6 weeks (second trial) of CrossFit training combined with GTE or PLA supplementation. Following warm-up cycling at 20 W, the participants continued cycling in 3-min stages. The starting power output was 40 W with progressive increases of 40 W each. The exercise was continued until exhaustion.

### Biochemical analyses

During each trial, blood samples from the antecubital vein were collected to test tubes with no anticoagulant or heparin tubes - first at rest and then at 3 min post exercise test and after 1-h recovery. Fresh whole blood samples were immediately assayed for reduced glutathione (GSH) by a colorimetric method with 5,5′-dithiobis-2-nitrobenzoic acid [24]. A portion of heparinized blood was centrifuged for 10 min at 1000 x g at 4 °C to separate plasma and erythrocytes that were then washed three times with cold saline (4 °C) and kept frozen at − 80 °C until analysis for activities of RBC antioxidant enzymes, i.e., superoxide dismutase (SOD, EC 1.15.1.1), glutathione peroxidase (GPx, EC 1.11.1.9), catalase (CAT, EC 1.11.1.6), glutathione reductase (GR, EC 1.6.4.2), plasma concentrations of total phenolics, uric acid (UA), lipid peroxidation products using the thiobarbituric acid (TBARS) reaction and ferric reducing ability of plasma (FRAP), and serum level of brain-derived neurotrophic factor (BDNF) as previously described [25]. The total antioxidant capacity of the GTE extract was assessed using the FRAP assay according to Benzie and Strain [26]. FRAP values were calculated from changes in absorbance at 593 nm at 37 °C to quantify the amount of colored ferrous tripyridyltriazine complex formed as a result of ferric to ferrous ion reduction at pH = 3.6. The final results were expressed in Trolox equivalents per g of GTE (μmol TE/g GTE). The total phenolics content of GTE were determined using the colorimetric method with phosphomolybdic-phosphotungstic acid reagent (Folin-Ciocalteu) adapted from reference [27]. The absorbance was read at 765 nm on a multi-mode microplate reader (Synergy 2 SIAFRT, BioTek, VT, USA), and the results are expressed as mg gallic acid (GAE) equivalents per g of GTE extract (mgGAE/gGTE).

### Statistical analysis

Data presented as means (± SD). A three-way repeated measures ANOVA with two groups (PLA and GTE) x two trials (at the start [1st trial] and at the end [2nd trial]) x three time points (at rest, 3 min post-exercise and post-1 h recovery) was followed, when appropriate, by the Bonferroni post-hoc comparisons to test for intergroup differences. With the aim of interpreting the results by indicating the relative degree to which the variance found in the ANOVA is associated with each of the main effects and their interaction, the Eta squared (η^2^), as a measure of the effect size in ANOVA, was calculated. Relationships between variables were evaluated using the Pearson’s correlation coefficient. Statistical analyses were performed with Statistica 10.0 (StatSoft, Tulsa, OK, USA). Statistical significance cutoff was *p* < 0.05.

## Results

Prior to the start of supplementation and CrossFit workout, maximal oxygen uptake (VO_2_max) was comparable for all study participants (Table 1, see Methods). Moreover, no significant intergroup differences regarding peak VO_2_max were found in the second trial.

**Fig. 1:**
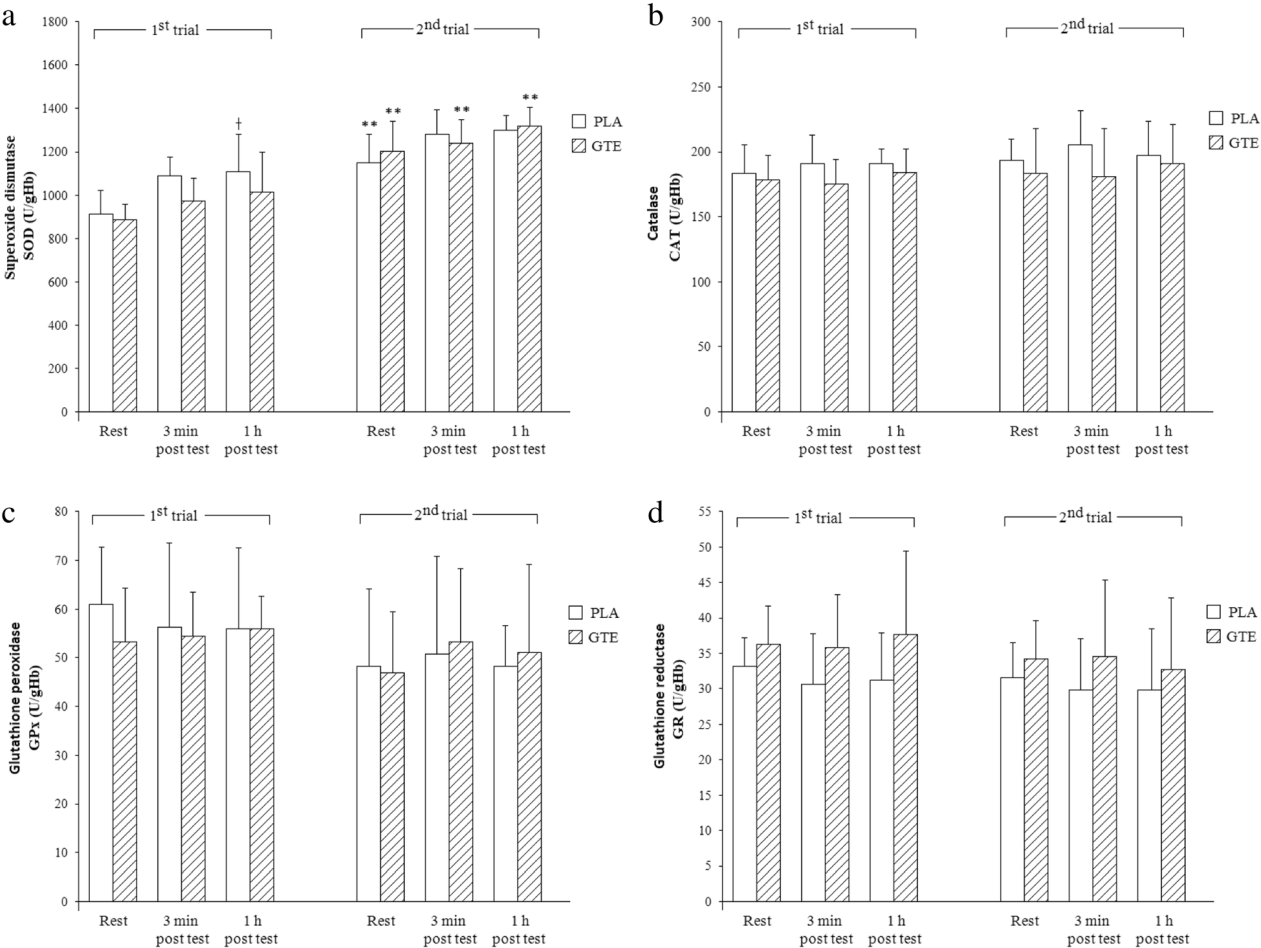
Activities of blood antioxidant enzymes in response to GTE supplementation and CrossFit workout; *Abbreviations*: *PLA* the control placebo group, *GTE* the supplemented with green tea extract group; *SOD*: Superoxide dismutase (**a**), *CAT*: Catalase (**b**), *GPx*: Glutathione peroxidase (**c**), *GR*: Glutathione reductase (**d**); Significant differences: **p < 0.001 vs. respective basal values before treatment; †*p* < 0.05 vs. resting values

Variables describing the blood enzymatic antioxidant defense are presented in Fig. 1.

Except for a significantly higher SOD activity (a) recorded at rest and post-exercise in both studied groups (PLA and GTE) in the 2nd trial compared to the corresponding values in the 1st trial, no significant differences were recorded among other assayed measures such as CAT (b), GPx (c), and GR (d). Moreover, in the 1st trial SOD, activity significantly increased post-1 h of recovery in the PLA group compared to the resting values. A repeated-measures 3-way ANOVA confirmed the significance of trial (F = 83.34, *p* < 0.0001; η^2^ = 0.86), time (F = 12.19, *p* < 0.0001; η^2^ = 0.47) and trial-group interaction effects on SOD (F = 7.91, *p* < 0.05; η^2^ = 0.36).

The results of biochemical analyses of the non-enzymatic antioxidant components such as GSH, UA and total phenolics concentrations are presented in Table 3.

**Table 3:** Concentrations of blood non-enzymatic antioxidants in response to GTE supplementation and CrossFit workout

Group	Variable	Trial	Rest	3 min post test	1 h post test
PLA	GSH (μg/mgHb)	1st	2.54 ± 0.2	2.34 ± 0.2	2.47 ± 0.3
		2nd	2.47 ± 0.4	2.39 ± 0.3	2.34 ± 0.3
GTE		1st	2.60 ± 0.4	2.59 ± 0.4	2.79 ± 0.54
		2nd	2.86 ± 0.3	2.76 ± 0.4	2.69 ± 0.3
PLA	UA (mg/dl)	1st	4.25 ± 1.3	4.82 ± 0.6	5.62 ± 0.9^†^
		2nd	5.63 ± 1.2^*^	5.96 ± 0.9^*^	6.83 ± 1.1^*†^
GTE		1st	4.93 ± 0.5	5.50 ± 0.8	6.62 ± 0.7^†^
		2nd	5.91 ± 1.0^*^	6.13 ± 1.1	6.82 ± 1.5
PLA	Total phenolics (g GAE/l)	1st	1.87 ± 0.08	1.76 ± 0.07^††^	1.74 ± 0.06^††^
		2nd	1.84 ± 0.05	1.73 ± 0.08^†^	1.72 ± 0.07^†^
GTE		1st	1.89 ± 0.10	1.79 ± 0.10^††^	1.73 ± 0.06^††^
		2nd	1.94 ± 0.07	1.84 ± 0.04^†^	1.79 ± 0.05^††^

Data are provided as mean ± standard deviation (SD); *Abbreviations: PLA* the control placebo group, *GTE* the supplemented with green tea extract group, *GSH* glutathione, *UA* uric acid; Significant differences: ^*^p < 0.05 vs. respective basal values before treatment; ^†^*p* < 0.05, ^††^*p* < 0.001 vs. resting values

Despite the lack of significant differences between the pre-and post-exercise GSH concentrations, a significant group effect (F = 5.05, p < 0.05; η^2^ = 0.27) was noted. There was a significant increase in plasma UA level during the recovery in both studied groups (PLA or GTE) with a significantly higher concentration in resting values in both groups, as well as in the post-exercise values in PLA in the 2nd trial. The significant trial (F = 9.15, *p* < 0.01; η^2^ = 0.39) and time (F = 35.84, *p* < 0.0001; η^2^ = 0.72) effects were revealed by 3-way ANOVA. In both groups, regardless of the trial, total phenolic components decreased after exercise. Moreover, there was opposite tendency towards a slight decrease in total phenolics in the PLA group as well as an increase in the GTE-supplemented group in the 2nd trial compared to the corresponding values in the 1st trial. The significant main effect of time (F = 76.38, *p* < 0.0001; η^2^ = 0.85) and closely related to the significant trial-group interaction effect (F = 3.57, *p* = 0.08; η^2^ = 0.21) were noted on blood total phenolics.

The next aim of the present study was to find out whether supplementation would affect FRAP, TBARS and BDNF concentrations (Table 4). Regardless of the supplement used (PLA or GTE) there was a significant increase in resting and post-exercise FRAP levels compared to the corresponding values in the 1st trial. The highest values of FRAP were recorded during recovery, regardless of the trial and the supplement used. Three-way ANOVA revealed significant effects of group (F = 11.78, *p* < 0.01; η^2^ = 0.46), trial (F = 85.15, *p* < 0.0001; η^2^ = 0.86), time (F = 61.65, *p* < 0.0001; η^2^ = 0.81) and trial-group (F = 11.10, *p* < 0.001; η^2^ = 0.44) interaction on FRAP. Plasma TBARS concentrations after six weeks’ CrossFit training depended on the study group: in the PLA group they were significantly higher, while in GTE-supplemented group they were slightly lower compared to the corresponding values before the start of the training. Moreover, there was a significant inverse correlation between FRAP and TBARS in the GTE-supplemented group (*r* = − 0.40, *p* < 0.05). Significant group (F = 27.40, *p* < 0.001; η^2^ = 0.67), trial (F = 10.35, *p* < 0.01; η^2^ = 0.43), time (F = 13.86, *p* < 0.0001; η^2^ = 0.49) and trial-group interaction (F = 43.07, *p* < 0.0001; η^2^ = 0.75) effects were revealed by 3-way ANOVA on TBARS. Although there were no marked differences between the groups, BDNF levels tended to increase immediately after exercise in both the PLA (*p* < 0.001) and GTE (no significant changes) groups as well as post 1 h of recovery in both PLA (*p* < 0.05) and GTE (no significant changes) groups, compared to values recorded at rest as reflected by a highly significant time effect (F = 13.73, p < 0.0001; η^2^ = 0.49). A six-week CrossFit workout resulted in slightly higher values of BDNF regardless of the group, as reflected by significant trial (F = 5.87, *p* < 0.05; η^2^ = 0.29) and trial-group interaction (F = 3.41, *p* < 0.05; η^2^ = 0.19) effects.

**Table 4:** Concentrations of plasma total blood antioxidant status, lipid peroxidation products and serum brain-derived neurotrophic factor in response to GTE supplementation and CrossFit workout

Group	Variable	Trial	Rest	3 min post test	1 h post test
PLA	FRAP (μmol TE/l)	1st	807.5 ± 39.2	865.6 ± 35.9	945.6 ± 46.0^†^
		2nd	1000.2 ± 75.5^**^	1036.7 ± 74.5^**^	1093.2 ± 61.8^**†^
GTE		1st	805.7 ± 108.2	887.8 ± 132.9	901.7 ± 156.3
		2nd	1166.1 ± 63.6^***^	1210.6 ± 57.5^***^	1308.1 ± 101.5^***††^
PLA	TBARS (μmol /l)	1st	4.72 ± 0.6	5.30 ± 0.5	4.65 ± 0.7
		2nd	6.28 ± 0.6^**^	6.98. ± 1.2^**^	6.19 ± 0.9^**^
GTE		1st	4.77 ± 0.7	5.16 ± 0.5	4.47 ± 0.7
		2nd	4.14 ± 0.6	4.92 ± 0.9	3.69 ± 0.6
PLA	BDNF (ng/ml)	1st	9.97 ± 2.6	12.24 ± 1.9^††^	11.47 ± 1.8^†^
		2nd	11.02 ± 2.6	12.00 ± 2.2	11.71 ± 1.4
GTE		1st	10.78 ± 2.4	11.73 ± 2.5	11.31 ± 2.2
		2nd	12.79 ± 2.1	13.19 ± 1.9	12.95 ± 1.7

Data are provided as mean ± standard deviation (SD); *Abbreviations: PLA* the control placebo group, *GTE* the supplemented with green tea extract group, *FRAP* ferric reducing ability of plasma, *TBARS* lipid peroxidation products, *BDNF* serum brain-derived neurotrophic factor; Significant differences: ^*^p < 0.05, ^**^p < 0.001,^***^p < 0.0001 vs. respective basal values before treatment; ^†^*p* < 0.05, ^††^*p* < 0.001 vs. resting values

## Discussion

The possible methods for attenuating oxidative stress and improving aerobic performance include dietary supplementation with natural phenolics and flavonoids [28] combined with regular physical activity [29]. Various studies have demonstrated that green tea is a potentially rich dietary source of antioxidant power [4, 30]. Benzie and Szeto [31] investigated in vitro antioxidant capacities of different types of tea including green tea. Phytochemical analysis has shown that the antioxidant activity of green tea measured by FRAP (Ferric Reducing/Antioxidant Power) assay, ranged from 699 to 1144 μmol TE/g of dry tea leaves, depending upon the brand of green tea. These data are comparable to our quantitative analysis, which revealed 1095 μmol TE/g GTE. The antioxidant activity of green tea extract is due to its relatively high content of polyphenolic compounds, including catechins. Our analysis confirmed the reliability of the amount of polyphenols declared by the manufacturer of the green tea extract used [30]. It should be noted that GTE extract contained only trace amount of caffeine (< 4 mg/kg). The participants from the GTE-supplemented group consumed two GTE capsules daily, which is equivalent to 0.1 mg caffeine/kg _body weight_, while a metabolic effect of caffeine is noticeable at 3–6 mg/ kg [32].

Given that a combination of aerobic and strength exercises during CrossFit workouts may lead to the improvement of aerobic and anaerobic endurance [18], our study showed an insignificant increase in VO_2_max changes (by only 4%) in the PLA group at the second trial compared to the corresponding values recorded at the first trial. Likewise, Murawska–Cialowicz et al. [20] reported that three months’ CrossFit training performed twice a week did not affect VO_2_max level in men.

Numerous studies confirmed the positive effect of GTE supplementation on aerobic performance in rodents [33] and in humans [11, 34–36]. To our knowledge this is the first study to evaluate the effects of medium-term GTE supplementation and CrossFit training on aerobic performance in young men. Our findings showed a marginal (7% vs. 4%) increase in VO_2_max in CrossFit-trained males receiving the GTE or the placebo capsules, respectively. Interestingly, tea catechins, especially epigallocatechin 3-gallate, combined with exercise could contribute to improvement in endurance capacity by promoting muscle oxygen consumption [11], mitochondrial biogenesis and fatty acid β-oxidation in skeletal muscle [33, 34] and whole-body fat utilization [35, 37]. An increase in aerobic capacity could be due to the synergy effect of catechins, caffeine, and other components contained of green tea extract [36].

The green tea catechins play an essential role in providing a balance between the generation of ROS and the endogenous antioxidant defense [38]. Therefore, the next aim of our study was to test whether GTE supplementation combined with CrossFit training would modify blood antioxidant capacity, which is classified by activities of antioxidant enzymes (SOD, CAT, GPx and GR) and concentrations of non-enzymatic antioxidants (GSH, UA and total phenolics). Six-week’s CrossFit training alone or combined with supplementation with green tea polyphenols at a dose of 490 mg led to a significant increase in SOD activity in both groups, but there were no changes in the activities of CAT, GPx, or GR. Superoxide dismutase is a major endogenous antioxidant enzyme, which catalyzes the dismutation of superoxide anion to hydrogen peroxide and molecular oxygen [39]. The increase in SOD activity in both groups (GTE and PLA) and a large effect of the training program (^2nd^trial vs. ^1st^trial) on SOD activity [ɳ^2^ = 0.86]) revealed by 3-way ANOVA, may imply important contribution of Crossfit training to stimulation of the cellular signaling pathways involved in up-regulation of superoxide dismutase as one of the main players in cellular antioxidant defense [29].

CrossFit training alone or combined with GTE supplementation did not significantly affect concentration of GSH, although a marginal (10%) increase in its level in the second trial in the GTE group was found (as reflected by a significant group effect). Our finding is consistent with previous studies on six-week *Ginkgo biloba* supplementation in physically active men [25]. It seems that dietary polyphenols could modulate the expression of γ-glutamylcysteine synthetase, the first enzyme of the cellular GSH biosynthetic pathway [40, 41]. Similarly, a six-week supplementation with GTE led to a marginal increase (2.6%) in total phenolic concentrations in the GTE group in the second trial, reflected by significant trial - group interaction effect. The chemical structure of polyphenols determines the rate and extent of absorption and the nature of metabolites circulating in the plasma; thus, the quantity of dietary polyphenols is not related to their bioavailability in humans [42]. Of note, maximum plasma concentrations for tea catechins were achieved at 2 h post-oral dosing and their low availability could be caused by slow absorption and wide tissue distribution [43]. Independent of the studied group (PLA or GTE), plasma total phenolics were significantly lower after completion of the graded exercise test (as reflected by a significant time effect and a large effect size), which implies that they were used as antioxidants against free radicals generated in contracting skeletal muscles.

One of the principal antioxidants in human plasma is uric acid - a terminal product of purine metabolism [44]. A significant increase in plasma UA level in response to graded exercise in both trials evidenced that intense exercise is a critical factor mediating increases in blood uric acid concentration. Moreover, lower resting changes in UA concentrations after a six weeks daily supplementation with GTE than placebo, expressed as percentage increase (namely 19.8% vs. 32%), suggest that green tea polyphenols can influence on increasing uric acid excretion [45].

Regular exercise may exert beneficial effects on plasma total antioxidant capacity and play an important role in the protection of the membranes against oxidative stress [46]. We observed that CrossFit workouts alone or combined with green tea supplementation resulted in a significant increase in plasma antioxidant capacity as measured by the FRAP method. What is noteworthy is that the relative FRAP changes in plasma, expressed as percentage increases, were twice as high after six weeks’ GTE consumption (24% for the PLA group and 44% for the GTE group). Tea catechins show powerful antioxidant capacities; they are localized near the surface of phospholipid bilayers of the membranes and are responsible for preventing oxidation of lipids exposed to oxygen radicals [47]. In the present study, a slight decrease in TBARS levels was observed after six weeks’ supplementation with green tea extract, which could have been caused by the interaction of green catechins with biological membranes. Moreover, Pearson’s rank correlation analysis revealed significant inverse interrelationships between concentrations of the FRAP and TBARS in the GTE group. On the other hand, a disposable physical load as well as a six-week CrossFit training had a significant impact on the increase in plasma TBARS concentration. Our data indicate that the higher antioxidant capacity provided by a combined activity of antioxidant enzymes and non-enzymatic antioxidants and substantially reduced blood marker of lipid peroxidation (TBARS) might be consequent to positive health outcomes induced by medium-term supplementation with GTE in CrossFit-trained men.

It is well documented that enhanced cognitive functions are connected with an increase in serum BDNF level, which is strongly expressed in the brain and, to a lesser extent, in the skeletal muscle, in order to stimulate synaptic plasticity and neurogenesis [48]. Previous reports critically point to antioxidant actions of polyphenols in the brain, because they reached extremely low concentrations in this tissue [49]. Gundimeda et al. [16] showed that green tea polyphenols (especially EGCG) in extremely low concentrations boost the neuritogenic activity BDNF, by the binding of EGCG to brain 67 kDa laminin receptor, leading to activation of NADPH-oxidase and generation of H_2_O_2_, which as a messenger influences the signaling pathway and therefore increases the neuritogenic ability of BDNF.

Our study did not demonstrate that a six-week CrossFit workout alone or combined with consumption of GTE markedly increased serum BDNF level. However, slight increases in serum BDNF level of around about 11 and 12% in the PLA and GTE groups, respectively, were observed (revealed by a significant trial-group interaction). Likewise, no significant increases in BDNF were revealed immediately post-test in both trials, regardless of the tested group (supported by a significant time effect). The insufficient increase of BDNF level can result from the fact that the subjects involved in CrossFit workouts were physically active before starting a six-week exercise program and serum BDNF baseline concentration was higher than in untrained men. A longer, three-month CrossFit training led to higher levels of BDNF in young physically active women and men [20].

The main limitation of our study designed to explore whether a medium-term consumption of green tea extract would improve blood antioxidant status and serum BDNF level in CrossFit-trained men is the lack of information on daily intakes of natural polyphenolic compounds included in their diet.

## Conclusions

Although six weeks’ consumption of green tea extract had only marginal effect on aerobic capacity and serum BDNF level in CrossFit-trained men, it caused a marked increase in the blood antioxidant capacity and moderate attenuation of the CrossFit-induced lipid peroxidation. Our results show that no general recommendations can be given concerning daily intakes of green tea extract by trained men and the choice of doses should be adapted to their individual needs.

## Abbreviations

AMRAP: As many rounds as possible
ANOVA: Analysis of variance
BDNF: Serum brain-derived neurotrophic factor
CAT: Catalase
EC: Epicatechin
ECG: Epicatechin-gallate
EGC: Epigallocatechin
EGCG: Epigallocatechin 3-gallate
FRAP: Ferric reducing ability of plasma
G: Gymnastics
GPx: Glutathione peroxidase
GR: Reduced glutathione
GSH: Reduced glutathione
GTE: The supplemented with green tea extract group
M: Metabolic conditioning
NADPH-oxidase: Nicotinamide adenine dinucleotide phosphate oxidase
PLA: The control placebo group
ROS: Reactive oxygen species
SOD: Superoxide dismutase
TBARS: Lipid peroxidation products
UA: Uric acid
VO_2_max: Maximal oxygen uptake
W: Weightlifting
η^2^: Eta-squared
